# Emerging Genotype-Phenotype Relationships in Primary Ciliary Dyskinesia

**DOI:** 10.3390/ijms22158272

**Published:** 2021-07-31

**Authors:** Steven K Brennan, Thomas W Ferkol, Stephanie D Davis

**Affiliations:** 1 Department of Pediatrics, Division of Allergy and Pulmonary Medicine, Campus Box 8116, Washington University School of Medicine, 660 South Euclid Avenue, St. Louis, MO 63110, USA; ferkol_t@wustl.edu; 2 Department of Pediatrics, University of North Carolina School of Medicine, 101 Manning Drive, Chapel Hill, NC 27514, USA; stephanie_davis@med.unc.edu

**Keywords:** primary ciliary dyskinesia, molecular genetics, genotype-phenotype association

## Abstract

Primary ciliary dyskinesia (PCD) is a rare inherited condition affecting motile cilia and leading to organ laterality defects, recurrent sino-pulmonary infections, bronchiectasis, and severe lung disease. Research over the past twenty years has revealed variability in clinical presentations, ranging from mild to more severe phenotypes. Genotype and phenotype relationships have emerged. The increasing availability of genetic panels for PCD continue to redefine these genotype-phenotype relationships and reveal milder forms of disease that had previously gone unrecognized.

## 1. Introduction

Motile ciliopathies are a group of disorders where motile cilia lining embryonic cells, airways, sinus and inner ear cavities, and ventricles in the brain are dysfunctional. The primary function of these hair-like projections is to facilitate the movement of fluid (i.e., mucus that lines the airways, cerebrospinal fluid) within various body cavities to be either recirculated or expelled [1]. Research into the structure and function of cilia in the last two decades has provided insights into the complex arrangement of the individual cilia components, and how genetic variation impacts component assembly and subsequent cilia function. This review will provide an overview of cilia structure and function, the genes involved in cilia assembly, and the phenotypic consequences of genetic variants in primary ciliary dyskinesia (PCD).

## 2. Overview of Primary Ciliary Dyskinesia

Presenting frequently in an autosomal recessive manner, PCD is a rare inherited disorder of motile cilia with an estimated incidence of 1:10,000 to 1:20,000 [2]. The condition typically presents early in life, with many infants experiencing respiratory distress at birth despite being born at term [3]. Symptoms of PCD are directly related to ineffective or absent beating of motile cilia in the embryonic node, respiratory, reproductive, and neurologic systems. This dysfunction results in impaired mucus clearance in the airways, abnormal sperm movement, or altered fluid flow in the fallopian tubes and ventricles within the brain. Affected people can present with a variable phenotype that can be severe or mild, leading to diagnostic challenges. Symptoms include chronic daily wet cough, chronic rhinorrhea, recurrent sinusitis, recurrent otitis media, bronchiectasis, infertility (or subfertility), situs abnormalities, and rarely hydrocephalus [4]. Sino-pulmonary symptoms are typically the most common manifestations of the condition. Approximately fifty percent of people with PCD will present with situs inversus, and heterotaxy is more common in this disease. Practitioners who see people with these complaints should have a high degree of suspicion for PCD so early identification and diagnostic testing can be performed.

## 3. Cilia Structure and Function

### 3.1. Overview

**Figure 1:**
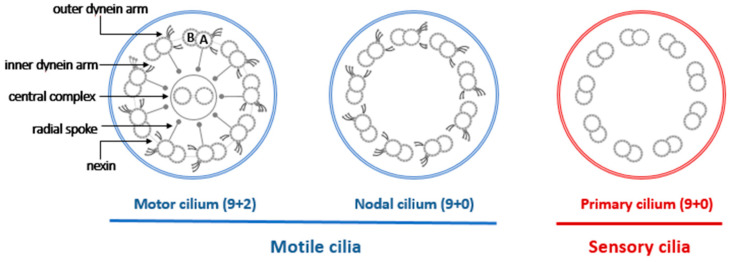
Diagram of basic cilia structure in 9+2 and 9+0 microtubule arrangements.

Cilia are organized structures of microtubules which protrude from the cell surface, serving many physiologic functions. Cilia are grouped into categories as motile or non-motile, and can have either a 9+2 or 9+0 arrangement of microtubule doublets (Figure 1) [1].

Motile cilia possessing the 9+2 arrangement are found primarily in the upper and lower respiratory tract, female reproductive tract, and spermatozoan. The primary function of motile cilia with the 9+2 arrangement is to move liquid and/or mucous parallel to the cells’ surface or in the case of spermatozoan flagellum, these cilia lead to the propulsion of the sperm. Motile cilia possessing the 9+0 arrangement are found in the embryonic node and play a primary role in determining left-right asymmetry of organs during human development by directing fluid flow across the cell surface [5]. This is achieved through a rotational motion of the cilium, which contrasts to the “wave-like” motion of the motile 9+2 cilia. Finally, primary cilia are usually solitary, nonmotile organelles that typically have a “9+0” microtubule configuration. Present on the surface of most non-dividing cells, these structures sense the extracellular environment. Additionally, they regulate developmental pathways, and defects lead to a growing number of complex clinically varied conditions. Affected individuals can have diverse features, such as variable mental disabilities, skeletal anomalies, obesity, retinal degeneration, and polycystic kidneys [6]. While overlapping conditions have been described, the role of these structures is not in fluid movement and thus are not directly implicated in the pathogenesis of motile ciliopathies [7].

### 3.2. Respiratory Cilia

Cilia lining the upper and lower respiratory tracts have a 9+2 configuration of microtubules that extend from the apical cell surface. Hundreds of these cilia are present on each ciliated cell and they move in a coordinated fashion, leading to effective mucociliary clearance or movement of mucus from the lower to the upper respiratory tract, out of the sinus ostia, or out of the middle ear space. The cilia structure is stabilized by hundreds of proteins whose alteration can produce the phenotype seen in PCD. A normally structured cilium shows an arrangement of nine microtubule doublets that are arranged along the circumference of the cilia along with another pair in the center referred to as the central apparatus or central complex. The outer doublets are anchored to the central complex by radial spoke proteins and anchored to one another by dynein and nexin proteins. This anchoring of microtubules by radial spoke and dynein-nexin complexes occurs very specifically, every 96 nanometers along the length of a cilium [8]. This prescribed repetition is key for generating an effective cilia waveform and motion.

The outer dynein arm is a motor complex which drives cilia beating [9], and is regulated by signals from the central apparatus and nexin-dynein regulatory complex, a group of proteins essential in determining cilia axonemal waveform [1]. Attachment of outer doublets to the central complex not only provides structure, but the central complex also relays mechanosensory signals to the outer doublet arms [10]. This information is essential for coordination of cilia motion and cilia lacking proper radial spoke protein assembly may have stiff and ineffective beating [11].

In addition to the proteins which make up the structural components of the cilia, there is a group of pre-assembly factors that are crucial to proper cilia assembly but reside in the cytoplasm and not in the cilia itself [12]. This large group is thought to play key roles in assembly of components in the cilia prior to their transport into a newly growing cilium. Basal bodies are an additional important structure in the growth of new cilia. The basal bodies consist of transition fibers necessary for intraflagellar transport and give rise to basal feet to help anchor the growing cilium [1]. Basal feet must retain a typical orientation to produce coordinated movement in multiciliated cells, and variation in proteins interacting with basal feet can alter this orientation and produce a PCD phenotype [13]. Genetic variants in pre-assembly proteins and their effects on cilia motion and effective beating is an area of ongoing research

## 4. Diagnosis

PCD is a complex and genetically heterogeneous disorder requiring multiple approaches for confirmatory diagnosis. There is not a single gold-standard test for the diagnosis of PCD. Two guideline statements outline potential strategies for clinicians [14,15]. The diagnostic toolkit includes medical history, transmission electron micocroscopy (TEM) for analysis of cilia ultrastructure, and genetic testing. Nasal nitric oxide (nNO) measurements can be a useful adjunctive test, provided cystic fibrosis has been excluded. However, reduced nNO levels alone are insufficient to make the diagnosis of PCD. People with motile ciliopathies who have mutations in some disease-associated genes can have nNO measurements that fall in a non-diagnostic range (Table 1 and Table 2). Availability of diagnostic tools also vary among sites, depending on the resources and experience of the institution. For instance, high-speed video microscopy is considered a diagnostic test in some European centers, but due to the lack of standardization and subjectivity, this tool is primarily used only on a research basis in the North America. Immunofluorescence staining of ciliated epithelial cells in vitro can also detect cilia abnormalities, and this technique is increasingly being used as a clinical diagnostic, replacing TEM in many European centers. However, it has not been widely adopted in North America.

## 5. Genotype and Phenotype Associations

### 5.1. Genotype Associations Based on Clinical Symptoms

Classic descriptions of PCD have focused on recurrent pulmonary infections and situs anomalies as the predominant presenting features. Four key clinical features characteristic of PCD have been reported and were incorporated in the American Thoracic Society clinical guidelines [87]. A positive answer to at least two of the four clinical features greatly increases the specificity and sensitivity of diagnosis. The features are: (1) unexplained neonatal respiratory distress in a term infant; (2) chronic wet coughing beginning at less than six months of age; (3) chronic daily nasal congestion beginning at less than six months of age; and (4) abnormalities of organ left-right asymmetry. Additional clinical features not listed but often present in PCD are chronic otitis media with middle ear effusion, and subfertility of both males and females owing to dysmotility of sperm or fallopian tube cilia.

Clinical features associated with specific genotypes may vary widely, leading to difficulties in identifying people with PCD. The severity of the manifestations associated with airway diseases can depend on the genetic variant encountered. People who have pathogenic variants in the genes *DNAH11* [20], *DNAH9* [18], and *RSPH1* [88] can have milder respiratory symptoms. In particular, people with biallelic *RSPH1* mutations have lower prevalence of neonatal respiratory distress, later onset of cough, and better lung function (FEV1) compared to age- and sex-matched cases.

Roughly half of people with PCD have situs abnormalities [89]. Although many components of the 9+2 arrangement in motor cilia are shared with the 9+0 arrangement in primary cilia, a genetic variant producing a phenotype in one type of cilia do not necessarily produce a phenotype in the other type of cilia. This is clearly illustrated in genes which encode structures integral for the development of the central complex and radial spokes. Since the 9+0 primary cilium does not have a central complex and radial spokes, it is not surprising that alterations in those genes do not affect nodal cilia development or function. Examples of this phenomenon include variants involved in structure of the radial spoke proteins encoded by *RSPH1* [88], *RSPH3* [35], *RSPH4A* [36,37,38], and *RSPH9* [36] and those in the central tubule pair such as *HYDIN* [25], *SPEF2* [24], and *STK36* [27]. Individuals with these genetic variants are not reported to have left-right laterality defects.

An important relationship between genotype and phenotype exists in the reproductive tract. The sperm flagellum possesses a 9+2 arrangement of microtubules and shares many proteins with motile cilia in the respiratory tract. In addition the luminal cells of the fallopian tube possess motile cilia to help move the ovum towards the uterus. There are reports of fertility for both males and females who have variants in outer dynein arm proteins encoded by *DNAH5* [50], *DNAH11* [21], and *CCDC114* [58], along with radial spoke proteins encoded by *RSPH4A* [21,36]. While these few patients illustrate the possibility of fertility in PCD patients, many people with PCD will experience infertility or subfertility [21].

### 5.2. Genotype Associations with Cilia Ultrastructrual Abnormalities

The variable association of clinical features with PCD genotypes also holds true for cilia ultrastructure. Transmission electron microscopy (TEM) provides an important tool in diagnosis of PCD, but cannot be relied upon as a gold standard due to the presence of normal cilia ultrastructure observed with many genetic variants [90] (Table 1). However, some genetic variants have obvious ultrastructural abnormalities (Table 2).

Microtubular disorganization associated with inner dynein arm and central apparatus defects are associated with variation in the genes *CCDC39* and *CCDC40.* The products of these two genes interact with one another and their proteins are intertwined in a braided fashion along the axoneme. They are critical for microtubule organization and accurate spacing of the inner dynein arm complexes every 96 nm [8]. Both gene products must be functional for normal cilia assembly, and a defect in one of these proteins cannot be compensated by a functional counterpart [80,91]. Notably, studies have shown that children who have biallelic mutations in *CCDC39* or *CCDC40* have nutritional deficits, greater lung disease, and more rapid pulmonary function decline when compared with individuals with other ultrastructural defects [80,91,92]. A brief report also suggested that both men and women who have these genetic and ultrastructural defects are more likely be infertile [21].

The most common cilia ultrastructure anomaly described is absence of the outer dynein arm. This finding is reported to occur in at least 20 of the ~50 published genes [92]. Structural proteins (DNAH5, DNAI1, DNAI2, NME8) [50,52,53,56], dynein arm docking complexes (CCDC114, CCDC151, ARMC4, TTC25) [57,59,61,62], and attachment factor CCDC103 [43] all routinely demonstrate absence of outer dynein arms. Cytoplasmic dynein assembly factor proteins (DNAAF1, DNAAF2, DNAAF3, DYX1C1, HEATR2) [69,71,73,76] have also been reported to have absence of outer dynein arms but this nearly always accompanies a loss of the inner dynein arm as well. Interestingly, the specific variant observed in a given gene can be responsible for different phenotypes. The p.Gly128fs25* variant of *CCDC103* produces a loss of the outer dynein arm, while the p.His154Pro variant has a variety of presentations ranging from normal ultrastructure to complete loss of outer dynein arms [64]. Research into variant specific phenotypes is an area of ongoing study.

Patients with radial spoke gene variants may have variable clinical presentations with mild or severe respiratory symptoms, and *situs solitus.* TEM alterations associated with *RSPH1* [88], *RSPH3* [35], *RSPH4A* [36], *RSPH9* [36], and *DNAJB13* [41] variants all show absence of the radial spoke apparatus, along with abnormalities in the central pair itself. The absence of radial spokes or central complex defects are not independently diagnostic for PCD, since these abnormalities have been found in the cilia of healthy control subjects. Clinical correlation and additional testing are recommended when encountering this ultrastructural finding [93]. While absence of radial spokes in respiratory cilia may produce a relatively mild phenotype, the absence of those same structures in the sperm tail is sufficient to lead to infertility [41].

There are a group of genes present in various parts of the cilium that when altered, produce no obvious ultrastructural abnormalities (Table 1). Examples of these include genes producing inner proteins of the microtubule (CCDC11) [42], nexin-dynein regulatory complex proteins (DRC1, GAS8) [29,33], and outer dynein arm proteins (DNAH9, DNAH11) [18,20]. While evaluation by transmission electron microscopy is an important diagnostic tool, the finding of normal or near-normal ciliary ultrastructure does not exclude the disease. Approximately 30% of people with PCD can have normal or near-normal axonemal structure [94].

### 5.3. Genotype Associations with Nasal-Nitric Oxide Values

Nasal nitric oxide is an important adjunctive test when evaluating people for possible PCD. This test has shown a reliable segregation of values when comparing individuals with PCD to those who do have this disease [95]. While this test is reported to have a sensitivity of 0.98 and a specificity of up to 0.99 in select populations, there remains a rare number of individuals diagnosed with PCD who retain a normal nNO value of >77 nL/min (Table 1 and Table 2). Generally speaking, genes which produce a more subtle clinical phenotype are associated with normal nNO values. Examples include *RSPH1* [88], *RSPH4A* [39], *RSPH9* [39], *DNAH9* [18], *GAS2L2* [13]*, HYDIN* [25], *CFAP53* [42], *NEK10* [43], *RPGR* [47], *STEK36* [27], *CFAP221* [26], *TTC12* [82], *CCDC103* [64], and *FOXJ1* [86]. Thus, while most people with PCD have reduced nNO levels, normal values do not exclude the disease, especially in people who have characteristic clinical features. A low nNO concentration warrants continued evaluation for PCD, provided cystic fibrosis has been excluded. It is also important to note that people with primary immunodeficiencies, conditions that have clinical features that overlap with PCD, can have reduced nNO levels and further testing may be needed [96].

### 5.4. Genotype Associations with Cilia Beating Abnormalities

A critical function of motile cilia in the respiratory tract is not simply their ability induce particle motion in the immediate area, but to do so in a coordinated fashion; thereby, leading to appropriate mucociliary clearance. Lack of effective cilia beat leads to decreased mucus clearance and the sequelae of recurrent lung infections, airway obstruction, and eventual bronchiectasis. Multiple genetic variations have been described which produce absent or ineffective beating patterns which are best assessed using high speed video microscopy (HSVM). One widely recognized pattern is the phenotype of the stiff and fast beating cilium. *GAS2L2* has been found to produce this phenotype with a beat frequency faster than baseline (19.8 Hz v 15.8 Hz) and ineffective coordination of the cilia due to abnormal cilia orientation [13]. Hyperkinetic and stiff beating cilia is also noted with those who have a variant in the outer dynein arm gene *DNAH11* [97]. A large number of proteins, notably those involved in cytoplasmic dynein assembly, have evidence of non-motile cilia when viewed under HSVM. The non-motile phenotype extends to other proteins involved in the outer dynein arms such as the outer dynein arm protein DNAH5 and intermediate light chain proteins DNAI1, DNAI2, [28]. This group has been noted to display both non-motile but also flickering cilium with beat frequencies that are less than 5 Hz, well below the frequency of a healthy control.

A third group of genes produce a decreased number of cilia on the cell surface. These genes are fundamental to ciliogenesis and genetic variation produces a classic PCD phenotype. Included in this group are *CCNO* [84] and *MCIDAS* [85] as genes which have been identified as important in the cell cycle and serve to differentiate airway stem cells into ciliated cells. Mutations in a key transcription factor that regulates cilia gene expression, *FOXJ1*, also reduce the number of motile cilia, and are clinically associated with hydrocephalus, recurrent respiratory infections, and laterality defects [86].

### 5.5. Genotype Associations with Non-Respiratory Syndromes

The overlap in proteins that underlie the structure of both motile and non-motile cilia creates the opportunity for overlapping of symptoms between various syndromes. A primary example of this is variants in *RPGR* where some individuals develop ocular symptoms, respiratory symptoms, and situs anomalies as a result of this shared protein product in ocular and respiratory cilia [48]. Neurologic manifestations such as intellectual disability, hydrocephalus or ventriculomegaly are increasingly seen in people with overlapping features of motile and primary ciliopathies. Individuals with one or more of these symptoms can have variants in the genes *OFD1*, *MCIDAS,* or *FOXJ1* [45,86,98]. Indeed, single mutations in *FOXJ1*, a transcription factor that regulates cilia gene expression, reduce the number of motile cilia, and clinically associated with hydrocephalus, recurrent respiratory infections, and laterality defects [86]. Individuals with homozygous loss of function variants in a recently reported gene, *TP73* have recurrent respiratory infections, bronchiectasis, and lissencephaly but interestingly do not have situs abnormalties [99].

Despite the large number of proteins shared between motile and immotile cilia structures, the overlap of neurologic, ocular, and respiratory symptoms remains rare. These overlapping syndromes are an area of ongoing research.

## 6. Conclusions

Primary ciliary dyskinesia is a rare, inherited disorder characterized by impaired ciliary function leading to chronic sinopulmonary disease, persistent middle ear involvement, laterality defects, and subfertility. During the past two decades, we have gained greater understanding of the genetic and pathophysiologic bases of PCD. Indeed, research has shown a clinical spectrum of motile ciliopathies, far broader that the classical form described by Kartagener nearly 90 years ago [100]. The development of powerful molecular biology and gene sequencing tools have allowed investigators to discover new genes, determine how encoded proteins are involved in ciliary structure and function, and produce model systems that have been invaluable in defining this disease. Along with the creation of multicenter clinical consortia, these tools have allowed us to identify and characterize various genotype-phenotype associations in PCD. While some of these relationships have yet to be fully defined, they serve as a general guide for clinicians and researchers who are trying to understand what has become an increasingly complex disease. While pathogenic variants in some genes can produce milder clinical symptoms, mutations in others produce severe clinical phenotypes and a potentially life shortening disease course. A high degree of suspicion and thorough evaluation utilizing multiple diagnostic methods are key to changing outcomes in PCD.

## Figures and Tables

**Table 1:** Genes, clinical features, and diagnostic measures described in people with primary ciliary dyskinesia and normal or near-normal ciliary ultrastructure.

Gene	nNO	Motility	Respiratory Symptoms	Laterality Defects	Subfertility
*DNAH1* [16,17]	NR	NR	Yes	Yes	Yes
*DNAH9* [18,19]	Low or Normal	Hypokinetic or reduced distal bending	Yes	Yes	Yes
*DNAH11* [20,21]	Low	Hyperkinetic	Yes	Yes	Yes
*DNAH17* [22]	NR	NR	NR	NR	Yes
*LRRC56* [23]	Low	Immotile, stiff, or twitching	Yes	Yes	NR
*GAS2L2***^c^** [13]	Low or normal	Hyperkinetic	Yes	NR	NR
*HYDIN* [21,24,25]	Low or normal	Immotile, rigid, rotational	Yes	NR	Yes
*CFAP221* [26]	Normal	Circular pattern	Yes	NR	NR
*SPEF2* [24]	Low	Stiff, rotational pattern	Yes	NR	Yes
*STK36* [27]	Normal	Stiff	Yes	NR	Yes
*CCDC164* [28,29]	Low	Stiff	Yes	NR	NR
*CCDC65* [30,31]	Low	Stiff, hyperkinetic	Yes	NR	NR
*GAS8* [21,32,33]	Low or normal	Normal or subtle beat abnormality	Yes	NR	Yes
*RSPH1***^a^** [21,34]	Low or normal	Reduced bending angle	Yes	NR	Yes
*RSPH3* **^a^** [21,35]	Low	Reduced bending angle	Yes	NR	Yes
*RSPH4A* **^a^** [21,36,37,38,39]	Low or Normal	Rotational pattern, stiff	Yes	NR	NR
*RSPH9* **^a^** [11,21,36,40]	Low or Normal	Rotational pattern, stiff	Yes	NR	Yes
*DNAJB13* [41]	Low	Reduced amplitude	Yes	NR	Yes
*CFAP53* [42]	Normal	Normal	Yes	Yes	NR
*NEK10***^b^** [43]	Normal	Normal	Yes	NR	NR
*OFD1* [44,45]	Low	Normal, immotile, or stiff	Yes	Yes	NR
NME5 **^d^** [46]	NR	NR	Yes	NR	NR
*RPGR* **^e^** [47,48]	Normal	Normal or uncoordinated	Yes	NR	NR

Normal nNO is >77 nL/min; NR—Not reported in literature; Footnotes: **^a^** Absent or defective radial spokes may be an acquired defect; **^b^** Shortened cilia; **^c^** Disoriented cilia; **^d^** Absent central pair; **^e^** X-linked transmission.

**Table 2:** The relationship between specific genes, ultrastructural defects, other diagnostic measures, and clinical features in primary ciliary dyskinesia.

Ultrastructure	Gene	nNO	Motility	Respiratory Symptoms	Laterality Defects	Subfertility
**Outer dynein arm defect**	*DNAH5* [21,49,50,51]	Low	Immotile or stiff	Yes	Yes	Yes
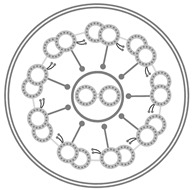	*DNAI1* [21,28,52]	Low	Reduced, minimal movements	Yes	Yes	Yes
	*DNAI2* [53]	NR	Reduced, minimal movements	Yes	Yes	Yes
	*DNAL1* [54,55]	Low	Immotile or weak	Yes	Yes	NR
	*NME8* [21,56]	NR	Normal	Yes	Yes	Yes
	*CCDC114* [57,58]	Low	Immotile or flickering	Yes	Yes	NR
	*CCDC151* [59]	NR	Immotile	Yes	Yes	NR
	*ARMC4* [28,60,61]	Low	Flickering	Yes	Yes	NR
	*TTC25* [62,63]	Low	Immotile or flickering	Yes	Yes	NR
	*CCDC103***^a^** [64,65]	Low or normal	Immotile or normal	Yes	Yes	Yes
**Outer and inner dynein arm defects**	*DNAAFI1* [21,66,67]	NR	Immotile	Yes	Yes	Yes
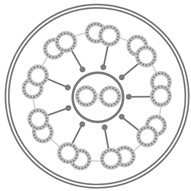	*DNAAF2* [68,69]	Low	Immotile	Yes	Yes	Yes
	*DNAAF3* [70,71]	Low	Immotile	Yes	Yes	NR
	*LRRC6* [21,68,72]	Low	Immotile	Yes	Yes	Yes
	*HEATR2* [73,74]	Low	Immotile or minimal movement	Yes	Yes	Yes
	*ZYMND10* [21,75]	Low	Immotile	Yes	Yes	Yes
	*DYX1C1* [21,68,76]	Low	Immotile	Yes	Yes	Yes
	*SPAG1* [21,77]	Low	Immotile	Yes	Yes	Yes
	*PIH1D3* **^a^** [78]	Low	Immotile	Yes	Yes	Yes
	*CFAP300* [68,79]	Low	Immotile	Yes	Yes	Yes
	*CFAP298* [30]	Low	Immotile	Yes	Yes	NR
**Inner dynein arm defect with axonemal disorganization**						
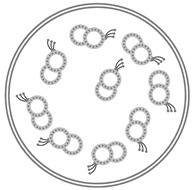						
					
					
					
	*CCDC39* [21,80,81]	Low	Immotile	Yes	Yes	Yes
	*CCDC40* [21,80,82]	Low	Immotile or stiff	Yes	Yes	Yes
	*TTC12* [83]	Low or Normal	Normal, immotile, or reduced beating angle	Yes	NR	Yes
					
					
					
**Oligocilia**						
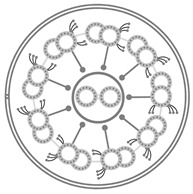						
					
					
	*CCNO* [21,60,84]	Low	Hypokinetic	Yes	NR	Yes
	*MCIDAS* [21,85]	Low	Immotile	Yes	NR	Yes
	*FOXJ1* [86]	Normal	Normal	Yes	Yes	Yes
					
					
					
					

Normal nNO is >77 nL/min; NR—Not reported in literature; Footnotes: **^a^** Mutation-specific.
